# Neural control of cerebral blood flow: scientific basis of scalp acupuncture in treating brain diseases

**DOI:** 10.3389/fnins.2023.1210537

**Published:** 2023-08-15

**Authors:** Guan-Yuan Jin, Louis Lei Jin, Bonnie Xia Jin, Jin Zheng, Belinda Jie He, Shi-Jiang Li

**Affiliations:** ^1^International Institute of Systems Medicine, Inc., Milwaukee, WI, United States; ^2^Ace Acupuncture Clinic of Milwaukee, LLC, Milwaukee, WI, United States; ^3^The Woodlands Acupuncture and Herbal Clinic, The Woodlands, TX, United States; ^4^Medical College of Wisconsin, Milwaukee, WI, United States; ^5^HCA Houston Healthcare Conroe, Conroe, TX, United States

**Keywords:** scalp acupuncture, cerebral blood flow, brain diseases, innervation of the scalp, core SA points or areas, neural stimulation, neural pathway, relative specificity

## Abstract

Scalp acupuncture (SA), as a modern acupuncture therapy in the treatment of brain diseases, especially for acute ischemic strokes, has accumulated a wealth of experience and tons of success cases, but the current hypothesized mechanisms of SA therapy still seem to lack significant scientific validity, which may not be conducive to its ultimate integration into mainstream medicine. This review explores a novel perspective about the mechanisms of SA in treating brain diseases based on its effects on cerebral blood flow (CBF). To date, abundant evidence has shown that CBF is significantly increased by stimulating specific SA points, areas or nerves innervating the scalp, which parallels the instant or long-term improvement of symptoms of brain diseases. Over time, the neural pathways that improve CBF by stimulating the trigeminal, the facial, and the cervical nerves have also been gradually revealed. In addition, the presence of the core SA points or areas frequently used for brain diseases can be rationally explained by the characteristics of nerve distribution, including nerve overlap or convergence in certain parts of the scalp. But such characteristics also suggest that the role of these SA points or areas is relatively specific and not due to a direct correspondence between the current hypothesized SA points, areas and the functional zones of the cerebral cortex. The above evidence chain indicates that the efficacy of SA in treating brain diseases, especially ischemic strokes, is mostly achieved by stimulating the scalp nerves, especially the trigeminal nerve to improve CBF. Of course, the mechanisms of SA in treating various brain diseases might be multifaceted. However, the authors believe that understanding the neural regulation of SA on CBF not only captures the main aspects of the mechanisms of SA therapy, but also facilitates the elucidation of other mechanisms, which may be of greater significance to further its clinical applications.

## Introduction

Scalp acupuncture (SA) is a modern acupuncture therapy that first emerged in the early 1970s, and has been widely used globally due to its significant efficacy on various acute or chronic brain diseases, specifically acute ischemic strokes (Lu, 1991; Zheng et al., 2011; Wang et al., 2012; Lee et al., 2013; Zhang et al., 2013; You et al., 2018; Yi et al., 2020; Huang et al., 2021; Xue et al., 2022; Wang J. H. et al., 2022).

In extensive clinical practice, several different schools of SA have emerged, some revise (mainly by adding) the earliest proposed SA locations (point, area, or zone) corresponding to the functional zones of the cerebral cortex (Jiao, 1997; Wang, 2019); others used classic meridians or acupoints distributed on the scalp as stimulation targets (Zhu, 2007; Xue et al., 2022); some have even integrated the hypothesis of biological holography (Molnar et al., 2018) to create their own unique systems of SA. Therefore, although there are many schools of SA, the primary mechanism explanations generally do not go beyond the above three categories. Of these categories, the meridian theory is considered as a primitive explanation in Traditional Chinese Medicine (TCM), the biological holography theory has not yet shown any biological evidence, while the hypothesis that certain SA locations correspond to the cortical functional zones has been the most attractive and accepted. This is because during body acupuncture, visceral diseases can often manifest as reflex points or zones on the body surface of the corresponding chest, abdomen, or back, based on neural reflexes at the same or nearby spinal segment (Jin et al., 2007). This can easily lead to a common misconception that SA locations have similar correspondence to the cortical functional zones. However, the cranial or occipital nerves innervating the scalp have no such segmental connections with the cerebral cortex. Furthermore, the stimulation of SA is mechanical rather than transcranial electromagnetic, making it impossible to stimulate the cortical functional zones directly below the scalp. Therefore, this hypothesis has been criticized (Ye et al., 2022) or opposed by statements such as “there is currently a lack of complete evidence for the mechanism of SA” (Wang et al., 2020).

This review explores a novel perspective about the mechanisms of SA in treating brain diseases based on its effects on cerebral blood flow (CBF). As supported by the authors’ pilot study (Li et al., 1997) and subsequent research applying functional magnetic resonance imaging (fMRI) techniques, CBF has been shown to be improved by acupuncture. Many brain diseases are linked to the reduction of CBF, while SA has been widely reported to treat such brain diseases and/or to improve CBF concurrently. Numerous clinical or laboratory studies have shown stimulation of the trigeminal, the facial, and the cervical nerves innervating the scalp area (including many core SA points or areas) may improve CBF. Accordingly, the authors propose that the efficacy of SA for brain diseases, especially acute ischemic strokes, is likely achieved by stimulating such nerves innervated on the scalp to improve CBF. Moreover, the possible neural pathways that produce these effects and the relative specificity of scalp stimulation at different locations in improving CBF are analyzed. The latter is closely related to the optimal selection of SA points or areas. The elucidation of the scientific basis of SA in treating brain diseases will undoubtedly further its clinical applications and raise its efficacy.

## Therapeutic effects of scalp acupuncture for brain diseases

Over the past 50 years, a large body of clinical evidence has been accumulated for the application of SA in the treatment of brain diseases, common cases including ischemic strokes, acute strokes, and post-stroke hemiplegia (Zheng et al., 2011; Wang et al., 2012, You et al., 2018; Huang et al., 2021; Wang Y.-F. et al., 2022). Others include autism (Yi et al., 2020), pediatric cerebral palsy (CP) (Xue et al., 2022), cognitive impairment (Zhang et al., 2013), and Parkinson’s disease (PD) (Lee et al., 2013).

Wang et al. (2012) conducted a meta-analysis of SA in the treatment of acute ischemic strokes including eight randomized controlled trials (RCTs) with 538 participants suffering from acute ischemic strokes. The results showed that SA significantly improved neurological deficit score and the clinical effective rate in the patients when compared with the control of conventional medicine (pharmaceuticals). Zheng et al. (2011) conducted a meta-analysis for evaluating the clinical outcome of SA in the treatment of acute intracerebral hemorrhage (ICH). Seven independent trials (230 patients) were included in this study. The results indicate that SA appears to be effective for improving neurological deficit scores in patients with acute hypertensive ICH and SA therapy for acute ICH is generally safe. You et al. (2018) conducted a meta-analysis to assess the clinical effectiveness of SA for stroke. Out of a total of 2,086 papers, 21 RCTs were selected. The results revealed that SA may significantly facilitate the recovery of motor and nervous functions in patients with acute to chronic stroke. A similar conclusion was reached from another meta-analysis by Huang et al. (2021), revealing that SA improves motor function in patients with post-stroke hemiparesis. Therefore, it can be said with confidence that SA has a good therapeutic effect on either acute or chronic stroke, regardless of brain infarction or ICH.

The efficacy of SA has also been well-scrutinized for many types of brain dysfunctions other than strokes. A recent meta-analysis involving 859 cases showed that SA is quite effective in dealing with autism (Yi et al., 2020). A meta-analysis consisting of 731 children of CP showed that SA was more effective than that of conventional rehabilitation in improving the symptoms of CP, and the procedure was deemed safe (Xue et al., 2022). Zhang et al. (2013) evaluated the therapeutic effect of scalp electroacupuncture (SEA) for mild cognitive impairment (MCI) in the early stage. A total of 233 MCI patients were randomly divided into three groups: the medication (nimodipine) group, the SEA group, and the syndrome differentiation group. Each patient was treated for two rounds (courses) of treatment, totaling 8 weeks. The results showed that while all three therapies improved the cognitive function of MCI patients, the efficacy rate of the SEA and syndrome differentiation groups were essentially equal, but both were far more superior to the medication group. In another RCT involving 64 infants with prenatal brain damage syndrome (BDS), Liu et al. (2016) observed that the developmental level of intelligence, motor function, linguistic, and social skills of these infants were enhanced by the intelligence seven-needle therapy that primarily stimulated Shenting (GV24), Benshen (GB13), and Sishencong (Ex.HN1) on the scalp.

Although the above studies all have significant effects of SA for various brain diseases, there are many research limitations, including smaller sample size, unclear stimulation parameters, etc.

## Potential mechanisms of scalp acupuncture for strokes

Over the recent years, many animal and clinical studies have been conducted on potential mechanisms of SA in treating brain diseases, especially strokes. According to current hypothesis of SA mechanism, its effect in restoring neurological dysfunction post-stroke is the result of stimulation of specific scalp areas in a reflex somatotopic system, corresponding to the functional zones of the cerebral cortex, with bioelectric effects transmitted to the cerebral cortex through meridians and nerves, thus altering the excitability of cerebral cortical nerve cells and accelerating the establishment of cerebral collateral circulation (Sun et al., 2020).

Certain clinical studies have also observed that applying SA on the region (ISSA_MS6) of the scalp direct above the cortical motor areas could induce vasodilatation of the cerebral blood vessels and better cerebral collateral circulation, raise CBF, lower the risks for infarction, and improve motor function for ischemic stroke cases (Zhang, 2003; Hsing et al., 2012). However, these effects also occur when a number of other scalp regions are stimulated, and it is not necessary to stimulate the specific scalp region corresponding to the cortical motor area. For example, multiple paralleled SA needles inserted at Baihui (GV20) and bilateral ISSA-MS8 could effectively increase the blood flow volume of the common carotid artery, leading to a rise of the energy supply of the cerebral blood circulation (Li et al., 2009). By functional magnetic resonance imaging (fMRI), it was observed that the contralateral somatosensory association cortex, the postcentral gyrus, and the parietal lobe were triggered when SA needles were inserted at the left Sishencong (EX.HN1), Chengling (GB18), Tianchong (GB9), and Jiaosun (TH20) of healthy volunteers (Park et al., 2009). In animal experiments with acute cerebral ischemia-reperfusion injury, it was observed that SA could suppress cytokines-mediated inflammatory reaction, attenuate cerebral ischemia-reperfusion injury, and improve neurofunctional rehabilitation (Zhang et al., 2007; Zhou et al., 2009).

Mechanisms of SA in the treatment of ischemic strokes are somewhat straightforward, especially in the acute stage, as SA stimulations may help reverse brain ischemia and improve neurological function by increasing CBF as it does for the facial nerve (Borsody and Sacristan, 2016). Several studies have shown that SA can prevent persistent thrombosis and increase vasodilation of the neurovasculature in the brain, and maintain blood circulation (Wang Y.-F. et al., 2022). By using fMRI diagnostics, among acute ischemic stroke patients, SA was shown being capable to enhance functional connectivity, particularly between visual, cognitive, motor control, and planning-related brain regions (Liu et al., 2020), or strengthen the functional activities related to sensory integration, language processing, and motor coordination of the dominant cerebral hemisphere and the motor control bilateral frontal lobe (Liu et al., 2021).

Of course, the mechanisms of SA in treating hemorrhagic strokes seems to be vastly different from the treatment of ischemic strokes. The main pathophysiological manifestations after ICH include early hematoma enlargement and perihematomal edema caused by catabolic products released from the hematoma. It was proposed that mechanisms of SA, such as on Baihui (GV20) penetrating Taiyang (EX.HN5) for the brain injury induced by cerebral hemorrhage may include increasing hematoma resorption rate, reducing cerebral edema, decreasing cerebral vascular permeability, and promote repairs for blood–brain barrier damage. It also may inhibit the inflammatory response in brain tissue around hematoma and improve the patient’s immune functions. Furthermore, it may modulate vascular function, prevention of additional brain injury, and the improvement of coordination and compensation function between cortical functional zones (Jiang et al., 2001; Bao et al., 2005; Dong et al., 2006; Liu et al., 2012).

However, because hypoperfusion or ischemia of surrounding brain tissue is also common in hemorrhagic strokes or traumatic brain injuries (TBI), resulting in hematoma (Sills et al., 1996; Zazulia et al., 2001; Schubert et al., 2009), the role of SA in the treatment of hemorrhagic brain diseases, especially its sequelae, cannot be separated from the role of improving CBF.

In short, from the above studies, the effect of SA improving CBF is one of most identifiable traits, and therefore has been frequently reported. It not only serves as the basis of various chronic or long-term effects of SA described previously, but is also most closely related to the instant efficacy of SA, to be described below.

## Instant effects of scalp acupuncture for stroke

Scalp acupuncture for stroke, whether hemorrhagic or ischemic, often has detectable instant effects, which means that in about 10 min after acupuncture, muscle strength in the paralyzed side could improve by 2 or more grades (Liu et al., 2012). Dong et al. (1994, 1996) found that 60.71% (34/56) ICH patients showed instant effect in the SA group, but no such luck in medication-therapy and surgery hematoma aspiration groups.

In an outpatient stroke rehabilitation unit, Molnar et al. (2018) conducted a prospective, assessor-blinded randomized control trial using Yamamoto’s New SA (YNSA), for 520 cases with post-stroke syndrome who had hemorrhagic or ischemic strokes and were admitted within 6 weeks of stroke. The results showed that in the YNSA group, all the sensory, motor, and functional scores improved significantly during the examination period until 3 years after injury. In some instances, effects were instantly observed only after a few minutes of SA, such as improved mobility of the limbs, with such effects lingering even for several weeks.

In a RCT, Wang et al. (2018) assessed the effect of SA on walking pattern of stroke patients, using three-dimensional gait analysis (3D-GA). The RCT was conducted for 30 patients in the subacute stage of ICH (1–3 months), all of whom were able to walk on their own. Participants were divided into two groups: SA (treatment group) or no intervention (control group). The treatment group received SA, a penetrating needle from Baihui (GV20) to Taiyang (EX.HN5), whose manipulation was repeated three times with an interval of 5 min. Shortly after the treatment, a significant difference was observed between the treatment and control groups in terms of spatiotemporal parameters of step length, speed and cadence and bilateral limb support. CBF has been found to be another key factor affecting gait performance (Gatouillat et al., 2015). Moreover, Inoue et al. (2002) also demonstrated that SA has a powerful and instant effect in eliminating limb paralysis caused by cerebral infarction or cerebral hemorrhage in rats.

The instant effect of SA in the treatment of strokes suggests that there must be a rapid response mechanism in the multifaceted mechanisms of SA. Because during the regulation of the physiological functions, only neural control has the characteristic of being rapid and accurate, it can be presumed that the instant effect of SA for strokes must also be achieved through rapid neural regulation. According to Liu et al. (2012), the mechanisms may be that SA improved the disorder of CBF in ischemic region, or that it changed the excitability of cerebral cortex nerve cell and led to the retroconversion of the excitability of cerebral nerve cell that was depressed by the stimulation of hemorrhage or the oppression of hematoma. SA has also shown to be capable of regulating the electrophysiological activity of brain nerve cells and changing excitability of cerebral cortex nerve cells, thus awakening brain nerve cells that were in the state of shock or dormancy after ICH (Wang et al., 2003).

The presence of the instant effect of SA not only illustrates the reliability of the curative effect of SA on acute strokes, but also suggests that its mechanism is definitely a rapid response. The improvement of CBF caused by SA is one of these rapid responses.

## Brain diseases related to reduced cerebral blood flow

Reduced CBF has been commonly described in brain aging and related neurodegenerative disorders, including Huntington’s disease (HD) (Chen et al., 2012), Alzheimer’s disease (AD) (Ruitenberg et al., 2005), PD (Chen et al., 2015; Pelizzari et al., 2019; Taguchi et al., 2019; Yang et al., 2021) and post-stroke sequelae (Hillis and Tippett, 2014), etc., suggesting that it is likely an important and early commonality in their otherwise distinct pathophysiological processes (Chen et al., 2012).

Several reports have suggested reduced CBF in HD, but little is known about the extent. Chen et al. (2012) used pulsed arterial-spin labeling MRI in conjunction with high-resolution anatomical MRI to non-invasively measure regional CBF (rCBF) in 17 early stage HD subjects and 41 healthy controls, and found profound yet heterogeneous CBF reductions in the cortex, extending to the sensorimotor, paracentral, inferior temporal, and lateral occipital regions, with sparing of the neighboring postcentral gyrus, insula and medial occipital areas. CBF in subcortical regions was also profoundly reduced, and to a similar degree.

The relationship between the severity of ischemic strokes and the infarct region is obvious. In fact, even during recovery from stroke, many sequelae symptoms are associated with inadequate CBF in certain brain regions. In a previous study that clinical interventions such as thrombolysis were used to treat acute ischemic strokes, Hillis and Tippett (2014) considered that patients with large areas of hypoperfusion beyond the infarct should be candidates for intervention to restore CBF. In most cases, the salvageable ischemic tissue is mainly confined to the cortex. The degree to which an individual recovers even simple cognitive functions is influenced by changes in CBF in the early period and by their degree of education level, as well as the intensity or level of initial severity of the stroke. Reperfusion of the distinct cortical regions of the left hemisphere, in the absence of infarction in that region, would restore the associated speech function. This suggests that during the rehabilitation, restoring CBF to specific cortical regions may yield improvements in certain symptoms associated with stroke-sequelae patients.

In fact, even in hemorrhagic strokes or TBI, a reduction of CBF also can occur. Schubert et al. (2009) observed that although all 17 patients with subarachnoid hemorrhage had intracranial pressure (ICP) and cerebral perfusion pressure within normal limits, they all had significantly reduced CBF. Those patients in better clinical condition had significantly smaller reductions in CBF than those with more severe hemorrhage, and had a comparably better prognosis. Changes in hypoperfusion were more pronounced in the supratentorial region (including the cerebrum) than in the infratentorial region (including the cerebellum).

Acute ICH also has shown substantial hypoperfusion zones around the hematoma that was interpreted as regional ischemia. Although there is no evidence for ischemia in the periclot zone of hypoperfusion in acute ICH patients studied 5–22 h after hemorrhage onset (Zazulia et al., 2001), substantial regions of reduced perfusion surrounding ICH might contribute to a substandard outcome and be amenable to anti-ischemic therapy (Sills et al., 1996).

Abnormal cerebral perfusion after TBI often leads to vasospasm. Maegawa et al. (2021) studied the relationship between the two in 25 patients, and found that the cerebral hypoperfusion of subarachnoid hemorrhage in the subacute phase could develop into post-traumatic cerebral vasospasm, thus emphasized the importance of aggressive treatment to prevent the development and progression of cerebral perfusion after TBI.

Other studies have also shown CBF is significantly reduced in patients with PD (Pelizzari et al., 2019) that correlates with the severity of the disease (Yang et al., 2021). In addition, previous studies have shown that the reduction in CBF induced by Madopar (levodopa benserazide) had a negative correlation with the Unified Parkinson Disease Rating Scale (UPDRS) scores, suggesting that improvement of symptoms was associated with increased CBF in the relevant areas (Chen et al., 2015).

All in all, there is apparent growing evidence that suggests various aspects of neuro-degenerative diseases are closely associated with the changes in cerebrovascular function (Yang et al., 2021). Changes in CBF could be used as important markers for disease diagnosis, mechanism investigation, and treatment assessment (Taguchi et al., 2019). It is theorized that treatments that improve or restore CBF may attenuate or possibly prevent the onset of these disorders (Sun et al., 2019). Therefore, as long as the SA can effectively enhance CBF, these brain diseases may be indicated for SA therapy. In fact, the clinical application of SA is most effective for patients suffering these types of brain diseases with significantly lower CBF, such as acute ischemic strokes.

## Effects of scalp acupuncture on cerebral blood flow

To date, numerous studies have shown that SA can increase rCBF, especially improving CBF disturbances in ischemic regions. This has been observed not only in healthy individuals (Byeon et al., 2011; Im et al., 2014) or animal experiments (Goadsby and Duckworth, 1987; Wang et al., 2017), but also confirmed during the treatment of some patients with brain diseases (Yang et al., 2021). Byeon et al. (2011) discovered that needling Baihui (GV20) increased CBF velocity in the middle cerebral artery (MCA) and anterior cerebral artery (ACA). Im et al. (2014) observed that needling Fengchi (GB20) in selected healthy male subjects increased CO_2_ reactivity in the basilar artery, but had no effect on the MCAs. Among other studies on the regulatory effects of acupuncture on CBF in PD, AD, and strokes, etc. (Li et al., 1999; Yong et al., 2009; Chen and Wu, 2011; Ratmansky et al., 2016; Kim et al., 2018; Ding et al., 2019; Yang et al., 2021), the stimulated locations included SA points or areas.

In a study consist of 15 PD patients with moderate symptoms, Yang et al. (2021) observed that needling Dazhui (GV14) and Fengchi (GB20) improved motor functions and subjective perception of the patients, while significantly increasing the total length of the internal carotid artery (ICA), the total length of the MCA, and the distal length of the M3 segment (that supplies blood to important components of the cortex – striatum circuit). From this, it seemed to be the mechanism by which acupuncture benefits PD patients. Besides, since the whole-brain CBF showed no significant difference before and after acupuncture, implying that acupuncture modulated the rCBF rather than increasing the whole-brain CBF.

Yong et al. (2009) observed in 30 patients of PD that SA combined with Madopar could improve the rigidity, tremor, dyskinesia and rCBF, indicating that the improvement of PD symptoms had a close relationship with the effect of SA on rCBF. Another study in the treatment of acute cerebral hemorrhage observed that the more significant the original CBF abnormality, the more significant the improvement after SA (Li et al., 1999).

Different SA points or areas from various parts of the scalp may produce different effects on rCBF, as such, the duration or intensity of acupuncture may also affect these effects. In a meta-analysis study, the effect of acupuncture was measured on the posterior circulation infarction vertigo (PCIV) that included 20 RCTs (1,541 participants) (Li et al., 2022). This study showed that acupuncture improved the vertebrobasilar blood flow velocity and achieved good efficacy for patients with PCIV. Moreover, longer duration of acupuncture interventions (more courses or sessions) and stronger stimulation (intensity) are generally more effective in improving vertebrobasilar blood flow velocity. For the acupoint selection, 33 main acupoints including SA points were used in the 20 studies, and Fengchi (GB20) was the most frequently used. Other researchers also indicated that needling Fengchi (GB20) may improve posterior cerebral circulation (Wang X.-X. et al., 2021; Wang Z.-Z. et al., 2021). Therefore, the use of Fengchi (GB20) for PCIV in clinical practice is highly recommended. Besides, Wu et al. (2017) observed the effect of long-term SA (treating for 5 months) on CBF in children with CP that SA increased the CBF, decreased the vascular resistance of ACA, MCA, and posterior cerebral artery (PCA), and improved the overall motor functions of the patients.

As mentioned above, the quantitative whole-brain CBF showed no significant difference before and after SA, implying that SA was modulating the distribution of cerebral blood supply. Therefore, in SA research or clinical practice, it may be better to observe and analyze the effect of SA on rCBF rather than the whole-brain CBF to show its impact on brain functions (Yong et al., 2009).

## Innervation of the scalp and its overlap and communication

The above-mentioned effect of SA on significantly improving CBF is clearly inseparable from stimulating the sensory nerves that innervate the scalp. Regarding the innervation of the scalp region, in brief, the anterior half of the scalp is dominated by the trigeminal nerve, while the posterior half of the scalp mainly receives occipital nerves from the cervical roots (C1∼C3) (Kemp et al., 2011).

The supratrochlear nerve (STN) and supraorbital nerves (SON) originate from the ophthalmic branch (V1) of the trigeminal nerve. The STN innervates the lower part of the forehead, while the SON innervates the skin from the forehead to the lambdoidal suture. The zygomaticotemporal nerve (ZTN), arising from the maxillary division (V2) of the trigeminal nerve, innervates the anterior portion of the skin in the temporal region. The auriculotemporal nerve (ATN), derived from the mandibular division (V3) of the trigeminal nerve, innervates the posterior portion of the skin in the temporal region.

The cervical nerves innervating the scalp include the greater occipital nerve (GON), the lesser occipital nerve (LON), the greater auricular nerve (GAN), and the third occipital nerve (TON). The grouping of these three nerves is also called the occipital nerve. The GON originates from the medial branch of the dorsal ramus of the C2 spinal nerve and provides cutaneous innervation to most of the posterior scalp regions as it travels up to the vertex. The LON originates from the ventral rami of spinal nerves C2 and C3, and innervates skin behind the ear. The GAN originates from the anterior branches of C2 and C3, and supplies the skin of the posterior ear and mandibular angle. The TON, the superficial medial branch of the C3 dorsal ramus innervates an area of skin just below the superior nuchal line.

It is of the utmost importance to note the possible overlap and communication between these sensory nerves. The overlap (also known as convergence) of nerve fibers refers to two or more nerves innervating the same scalp area (Tubbs et al., 2011; Capek et al., 2015), which mainly occurs at the junction of adjacent or bilateral innervation areas (such as at the midline of the scalp). It was reported that small branches were found to cross the midline and communicate with the contralateral TON inferior to the inion in 66.7% of patients (Tubbs et al., 2011). In contrast, communications between neurons refers to the synaptic connection between two or more neurons (Lovinger, 2008). It is known that all three occipital nerves (GON, LON, and TON) located in the posterior neck and scalp regions are interconnected through their communicating branches regularly (Kemp et al., 2011).

Therefore, by needling an acupoint or area with overlaps of innervations, afferent stimulation information can be transmitted in parallel through different nerves. When needling an acupoint or area filled with communication, afferent stimulation information is transmitted, often through synaptic connections. Presumably, the stimulation amount of acupuncture or the degree of induced effects would be different from that of the stimulation of a single innervation area.

Many areas on the scalp have overlap or communication of different innervating nerves, such as between the trigeminal nerve or the GON distributed on either side of the midline; between two or more adjacent nerves distributed on the front and back of the same side of the scalp [such as between the ATN and the temporozygomatic nerve (TZN), the SON, or the GON or the LON, etc.]. In fact, in some scalp areas, due to the close proximity of two different nerves, even if they do not overlap or communicate, they may be successively stimulated with a single needle (such as 1-inch horizontal needling) (Li et al., 1999) inserted into the subcutaneous area at a certain distance. If SEA is applied, the propagation of the current is impossible to be limited to the stimulation of a single nerve.

Herewith, it is also important to note another type of overlap, or strictly speaking, a convergence mechanism that could occur after two sensory afferents enter the brain (Piovesan et al., 2003), as in the secondary sensory neurons of the trigeminal nerve. It also affects the specificity of the effects of SA when stimulating different nerves. In addition, the frontal and occipital muscles on the scalp are innervated by the superior zygomatic nerve (SZN) of the facial nerve and the posterior auricular nerve (PAN) (Yu and Wang, 2022), respectively, so that when stimulating the frontal or occipital areas with SA, it will also stimulate these two branches of the facial nerve besides stimulating the trigeminal nerve or the occipital nerve, respectively. It is currently known that in the face, there are dense and rich communication connections between the trigeminal nerve and facial nerve (Hwang et al., 2015).

## The core scalp acupuncture points or areas for brain diseases and their innervation

Recently, a systematic review and meta-analysis was conducted by Wang Y.-F. et al. (2022) where the researchers extracted 33 SA points or areas from 35 RCT studies of SA for post-stroke hemiparesis. The study analyzed the SA point-selections data by applying an association rule based on the *Apriori* algorithm. The results showed that Baihui (GV20), Shenting (GV24), Yintang (Ex.HN3), and ISSA_MS6_i (ISSA Anterior Oblique Line of Vertex-Temporal, Lesion-Ipsilateral), ISSA_MS7_i (ISSA Posterior Oblique Line of Vertex-Temporal, Lesion-Ipsilateral), ISSA_PR (ISSA Parietal Region, comprised of ISSA_MS5, ISSA_MS6, ISSA_MS7, ISSA_MS8, and ISSA_MS9) (WHO Scientific Group, 1991; Liu et al., 2012) could be considered as the core SA location-combos in the treatment of post-stroke hemiparesis.

In daily SA clinical practice, the modern stimulation locations of SA are often labeled with meridian-based acupoints well-known to most acupuncturists (Wang et al., 2018). For examples, ISSA_MS5 is the connecting line from Baihui (GV20) to Qianding (GV21), along the vertex midline; ISSA_MS6 is the anterior oblique line of vertex-temporal from the anterior one of Shishencong (Ex.HN1) obliquely to Xuanli (GB6); ISSA_MS7 is the posterior oblique line of vertex-temporal from Baihui (GV20) obliquely to Qubing (GB7).

In a database of acupoints for PD established by Li et al. (2020), 184 acupoints, across 168 eligible papers, were selected. These points were mainly distributed in the head and neck areas and at the extremities. Among them, Taichong (LR3), Baihui (GV20), Fengchi (GB20), Hegu (LI4) and Chorea-Tremor Controlled Area were the most frequently used acupoints/areas. In a separate study to evaluate the effects of acupuncture on vascular dementia (VD) by Feng et al. (2015), 238 acupuncture prescriptions were included for analysis. Baihui (GV20), Sishencong (EX.HN1), Fengchi (GB20), Shenting (GV24), and Shuigou (GV26) of the scalp were the most frequently used acupoints for treatment of VD.

Yu et al. (2018) conducted a correlation analysis on the data of acupuncture prescriptions in the clinical literature regarding acupuncture treatment for AD, and observed the high-usage of 15 acupoints. Among them, those located on the scalp are Baihui (GV20), Sishencong (EX.HN1), Fengchi (GB20), and Shenting (GV24). Out of these, Baihui (GV20) seems to be the most frequently selected acupoint for AD. The intelligence seven needle therapy used by Liu et al. (2016) for infants with prenatal BDS primarily stimulates Shenting (GV24), Benshen (GB13), and Sishencong (Ex.HN1).

Since modern anatomical studies have been completed on almost all the acupoints of the body, including the scalp (Yan, 1983), one can easily label the core SA points or areas using the scalp nerves as follows: the frontal nerve originating from the V1 can be divided into the STN and the SON. The STN innervates Shenting (GV24), which is at 0.5 inches directly above the midpoint of the anterior hairline. SON innervates Benshen (GB13), which is on the lateral part of the forehead, 0.5 inches within the anterior hairline, 3 inches lateral to Shenting (GV24), in the frontalis muscle; Baihui (GV20) is primarily governed by the GON and the frontal nerve. Sishencong (Ex.HN1) has branches of the GON, ATN, and SON; Fengchi (GB20) is distributed with branches of the LON; Fengfu (GV16) is distributed with branches of the TON and the GON; there are branches of the GON in the ISSA_MS13 (Optic Area) and ISSA_MS14 (Balance Area) located on both sides of the midline of scalp. The ATN and the SON innervate the Chorea-Tremor Controlled Area, located on a parallel line, 1.5 cm in front of ISSA-MS6.

Among the points used from the YNSA method, the Parietal Acupoints (PTAT) are located on the midline from 5 cm anterior of the coronal suture to over the lambdoid suture, with a 2-cm width on the scalp (Aoyama et al., 2017). This area almost completely overlaps with the segment of GV on the vertex, including the ISSA_MS5 and Sishencong (EX.HN1). Their anterior and posterior parts are innervated by the trigeminal nerve and GON, respectively. Moreover, the Ypsilon points are distributed on the anterior and posterior sides of the auricle, in both sides of the scalp, innervated by the ATN and the LON.

**FIGURE 1:**
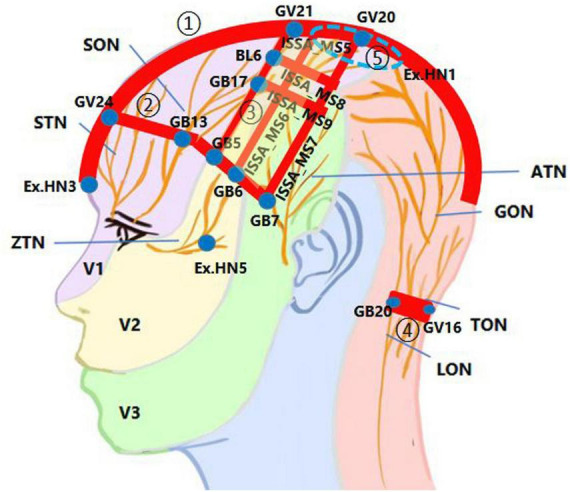
The innervation in the core scalp acupuncture (SA) points or areas used for brain diseases. The scalp nerve distribution patterns were redrawn based on the inspiration of a figure in Khansa et al. (2016). ATN, auriculotemporal nerve; GON, greater occipital nerve; LON, lesser occipital nerve; SON, supraorbital nerve; STN, supratrochlear nerve; TON, third occipital nerve; V1, ophthalmic branch of the trigeminal nerve; V2, maxillary branch of the trigeminal nerve; V3, mandibular branch of the trigeminal nerve; ZTN, zygomaticotemporal nerve. ① The midline area from Yintang (Ex.HN3) to above the lambdoid suture, with a 2-cm width on the scalp (red band). ② The region between Shenting (GV24) and Benshen (GB13) along the anterior hairline (red band), that includes ISSA_MS1, ISSA_MS2, ISSA_MS3, etc. ③ The region is surrounded by Baihui (GV20), Qianding (GV21), Xuanlu (GB5), Xuanli (GB6), and Qubin (GB7), which is a horizontal trapezoid composed of ISSA_MS5, the Chorea-Tremor Controlled Area, the lateral hairline, and ISSA_MS7 (light yellow box). ④ The region between Fengchi (GB20) and Fengfu (GV16) along the posterior hairline (red band). ⑤ The EX.HN1 region is surrounded by the four points of EX.HN1 (blue dash circle).

Figure 1 shows the core SA points or areas frequently used for brain diseases, with the distribution of scalp nerves referenced in Khansa et al. (2016). Because acupoint is an area instead of a spot (Jin et al., 2007), in this figure, solid blue dots represent acupoints, while red bands represent the connecting areas between them or those of the international standard scalp acupuncture (ISSA) system (WHO Scientific Group, 1991); five particular SA regions are remarked by circled Arabic numbers.

From this figure, the distribution of core SA points or areas have at least three key features: 1. Centered around Baihui (GV20) with the scalp segment of the GV as the main axis. 2. Centered around the boundary between the front and back halves of the scalp along the oblique line from the vertex to the front of the auricle, especially the posterior edge of the front half. 3. Centered along the anterior, lateral and posterior hairline.

From the perspective of scalp innervation, most of these locations, such as ISSA_MS5, ISSA_MS6, ISSA_MS7, ISSA_MS8, ISSA_MS9, the Chorea-Tremor Controlled Area, and those SA points or areas along the frontier hairline, are located in the region innervated by the trigeminal nerve, or at the overlap of the bilateral same nerves or several different nerves on the same side of the scalp. This may also be the main neural basis for stimulating these areas with strong needle sensation or significant therapeutic effects. Of course, certain branches of the facial nerve innervating these areas may be stimulated simultaneously. Actually, 11 of the 14 ISSA lines (WHO Scientific Group, 1991) are distributed on the front half of the scalp innervated by the trigeminal nerve. Although Yintang (Ex.HN3) is not located on the scalp, it is still categorized as part of the SA points because it extends from Shenting (GV24) and also is innervated by the V1 of the trigeminal nerve. The significance of stimulating the trigeminal nerve with SA can be attributed to the direct axonal reflex pathway between the sensory afferent of trigeminal nerve and certain intracranial arteries (White et al., 2021).

However, it should be noted, however, that these so-called “core SA points or areas” were selected mainly on the basis of their high frequency of application in the related literature to date, without comparison to other SA points or areas in terms of efficacy. Their high frequency of application is also due to the current hypothesis of the SA mechanisms, which speculates the role of these points or areas corresponding to the cortical functional zones. Therefore, further research is warranted to determine whether they are the most effective stimulation locations for treated brain diseases. Moreover, the frequently used SA points or areas are not limited to locations shown in Figure 1.

## Stimulating the trigeminal, facial, and cervical nerves to improve cerebral blood flow

Cerebral blood flow is usually determined by cerebral perfusion pressure and cerebrovascular resistance, while the latter itself depends on the degree of vasodilation and blood viscosity (Fantini et al., 2016). The brain relies mainly on changes in vascular caliber and systemic arterial pressures to maintain CBF (ter Laan, 2014). In the recent decades, many experiments or clinical trials are beginning to investigate the impact of CBF under direct stimulation of the trigeminal nerve or facial nerve innervating the scalp (or head and face).

It is known that the trigeminal nerve innervates the majority of the cerebral arteries and significantly contributes to the control of cerebrovascular tone in both healthy and diseased states (White et al., 2021). Upon stimulation of the trigeminal nerve, it permits direct modulation of CBF (Li et al., 2021). Research has shown that stimulation of the trigeminal nerve may be topographically oriented, with specific branches (the V1, V2, and V3) leading to an increased CBF in specific regions of the intracranial arterial tree (White et al., 2021).

Most intracranial vessels and dura mater, as well as the prefrontal skin are innervated by the ophthalmic branch of the trigeminal nerve. In some healthy subjects, both studies by Suzuki et al. (2020) and Waki et al. (2017) found that electroacupuncture at the V1 of the trigeminal nerve enhanced CBF in the prefrontal cortex. The nasociliary nerve, which originates from the V1, contains major vasodilator innervation for the MCA, and its stimulation results in the release of vasoactive neuropeptides in the MCA region (Atalay et al., 2002). Li et al. (2019, 2021) in recent studies observed that stimulation of the infraorbital nerve of the V2 dilates the ICA and that such vasodilatory response of cerebrovasculares is retained after SAH-induced cerebral vasospasm. The lingual nerve (LN) from the V3 of the trigeminal nerve is known to innervate the ICA. Ishii et al. (2014) demonstrated that stimulation of the LN leads to an enhanced CBF within the ICA. When considering the geographic distribution of the trigeminal nerve in the face, a possible distinction exists between different branches of the trigeminal system and the areas that they innervate, and this geographic specificity has the potential to guide a more specific treatment of trigeminal nerve stimulation (Li et al., 2021).

The facial nerve is a mixed nerve composed of motor, sensory, and parasympathetic fibers. To date, numerous studies have shown that stimulation of the parasympathetic fibers of the facial nerve rapidly dilates the cerebral arteries and increases CBF, no matter if that stimulation is delivered at the facial nerve trunk or at distal points such as the sphenopalatine ganglion (SPG, or called pterygopalatine ganglion, PTG). Generally, facial nerve stimulation increases CBF in a manner that it is deemed sufficient in reversing brain ischemia and improving neurological function (Borsody and Sacristan, 2016).

The facial nerve is the efferent limb in a cerebrovascular reflex with one or more sensory afferent limbs (e.g., trigeminal nerve). In the brainstem reflex evoked by stimulation of the face, the sensory endings of the trigeminal nerve are excited first in response to tissue damage, while the facial nerve might serve as the effector (Borsody and Sacristan, 2016). Therefore, the significance of stimulating the facial nerve in a clinical setting is to provide means to counteract brain injuries. For example, it could be used as an emergency treatment for conditions of brain ischemia such as ischemic strokes. Presently, several methods have been developed to activate the facial nerve, including non-invasive medical devices to give the facial nerve or the geniculate ganglion region magnetic stimulation through coil devices, placed on either side of the head or the ears (Borsody and Sacristan, 2016; Baker et al., 2021).

The effect of stimulating the occipital nerve on CBF was observed mainly by stimulating the cervical medulla of experimental animals. For example, stimulation of the cervical medulla in rats leads to cerebral vasodilation and a significant increase in cortical CBF (Sagher and Huang, 2000). This similar result was also observed in humans, and the ability to stimulate the spine to increase CBF is peculiar to higher cervical medullary stimulation with moderately low frequencies (Hosobuchi, 1985; Isono et al., 1995). Since the nerves innervating the posterior scalp mainly originate from the C1-C2 of the higher cervical medulla, there are many studies in recent years on the effect of SA on CBF, in which Fengchi (GB20) innervated by the LON was stimulated; most of these studies observed the improvement of the cerebral posterior circulation (Dong et al., 2020; Wang X.-X. et al., 2021; Wang Z.-Z. et al., 2021; Li et al., 2022).

The above-mentioned changes in CBF observed by direct stimulation of the trigeminal, facial nerves, etc. can satisfactorily explain why similar results of SA improving CBF are commonly observed in clinical practice. In other words, the improvement in CBF resulting from different SA points or areas is achieved by stimulating the corresponding nerves of the scalp.

## Mechanisms of stimulating scalp nerves to improve cerebral blood flow

Mechanisms of stimulating scalp nerves to improve CBF can be explored by reviewing the structural characteristics of cerebral circulation with its neuromodulation. The blood supply to the brain tissue is accomplished by the ICA system and the vertebrobasilar artery system, the latter also known as the posterior circulation system, composed of the vertebral artery, basilar artery, and their branches. The brain is one of the most metabolically demanding tissues in the body, thus the cerebral arteries have several specificities that help differentiate themselves from other arteries in the body in order to meet the high demand for blood flow.

First, the “Circle of Willis” is formed from several interconnecting arteries, including the PCA, the posterior communicating artery, the ACA and the anterior communicating artery, In humans, CBF is mainly provided by the ICAs whereas the vertebral and spinal arteries supply the brainstem. The unique blood vessel organization of the “Circle of Willis” acts as a distribution center permitting blood to flow in any direction to meet increased demand and overcome stenosis (Roloff et al., 2016). When one of the arteries is blocked or narrowed, blood from the health side flows compensatory to the ischemic area, alleviating or eliminating the symptoms caused by the vascular blockage or stenosis.

Second, a unique characteristic of cerebral arteries is that they are both conduits and resistance vessels that are unlike any other arteries that constrict and dilate. In other words, the contraction and dilation of cerebral blood vessels have an autoregulatory capability (Faraci and Heistad, 1990), which protects the brain from sudden rises in blood pressure to prevent stroke, whereas vasodilatation can prevent ischemia (Roloff et al., 2016).

Third, cerebral arteries differ from peripheral arteries in another major way: they are richly innervated with neuronal fibers of parasympathetic origin, providing the most powerful vasodilatory mechanism (Goadsby, 2013). Parasympathetic fibers follow the sympathetic nerves along cerebral vasculatures (Duckles, 1981). The parasympathetic fibers innervating the anterior circulation of the cerebral artery are mainly derived from four parasympathetic ganglia: they are SPG, cavernous ganglion (CG), otic ganglion (OG), and carotid mini ganglion (CmG). Although there is some evidence for parasympathetic innervation of the vertebrobasilar arteries, their origin remains unknown (Roloff et al., 2016).

Fourth, cross talk between parasympathetic and sympathetic nerves exists in the cerebral arterial wall. This is because parasympathetic and sympathetic nerves usually are intertwined on cerebral arteries or run parallel within the same perineural sheath. The axo-axonal contact distance between them can be as low as 25 nm, compared to the 100 nm commonly seen for neuromuscular contacts in the same vessel. This close apposition suggests cross-talk between parasympathetic and sympathetic nerve fibers (Roloff et al., 2016). It has been observed that during a “fight or flight” situation where there is an increased sympathetic drive, the vertebrobasilar arteries may vasodilate (rather than constrict) via the mechanism to ensure the brainstem and/or cortex remain well perfused at times when peripheral organs experience vasoconstriction and reduced vascular conductance (Lee et al., 2011).

Fifth, the cerebrovasculares are innervated by sympathetic, parasympathetic, and sensory nerve fibers, all of which play an important role in cerebrovascular regulation. The trigeminal nerve is the largest cranial sensory nerve, with three bilaterally paired branches that spread across the face. In the brain, the trigeminal nerve converges at the trigeminal nucleus and has known connections to various regions of vasoregulatory control. Outside the brain substance, the trigemino-cerebrovascular network, which originates from the V2 and V1, innervates most of the cerebral vasculature system, including the large arteries, pial vessels, and venous sinuses. Therefore, stimulation of the trigeminal nerve can directly modulate CBF via these networks (Li et al., 2021). Those sensory nerve fibers that can affect cerebrovascular tone are called “sensory-motor nerves” (Rubino and Burnstock, 1996) and contain substance P and calcitonin gene–related peptide (cGRP) (Roloff et al., 2016).

Based on the above characteristics of the cerebral vascular structure, stimulation of the parasympathetic nerve fibers innervating the cerebral vasculature results in an increase in CBF accompanied by cerebral vasodilation; while stimulation of the sympathetic nerves innervating the cerebral vasculature may experience brief vasoconstriction, the subsequent effects are mostly increases in CBF.

It is known that there are two sources of innervation controlling blood flow to cerebrovasculares: extrinsic and intrinsic. The intrinsic innervation originates from local neurons [γ–aminobutyric acid (GABA)–ergic interneurons in the cerebral cortex (Suzuki et al., 2020)] within the central nervous system targeting arterioles to control blood flow in the brain parenchyma. The extrinsic innervation from peripheral ganglia innervate all major cerebral arteries before they enter the brain parenchyma (Roloff et al., 2016).

Extrinsic neuromodulation of CBF is mainly involved in the primary sensory neurons of the trigeminal nerve and postganglionic fibers of the facial nerve. They cause cerebral vasodilation by releasing different neurotransmitters: primary sensory neurons of the trigeminal nerve induce vasodilation by releasing cGRP, substance P, and pituitary adenylate cyclase-activated peptide. Facial nerve postganglionic fibers originate from the SPG and OG induce vasodilation by releasing acetylcholine (ACh) and vasoactive intestinal peptide (VIP). Originating in the superior salivatory nucleus, the greater petrosal nerve triggers vasodilation by synapsing with nitric oxide (NO)–ergic neurons in the SPG, which causes them to release NO from their terminals. In addition, the dural arteries are innervated by postganglionic fibers from the facial nerve, which originates from trigeminal primary sensory neurons and the SPG; trigeminal stimulation may increase blood flow in the dural arteries via an axonal reflex mechanism (Suzuki et al., 2020).

Stimulation of the trigeminal nerve at least has a threefold effect on cerebral vasculature, with each individual action leading to increased CBF (Suzuki et al., 2020): (1) The antidromic pathway: stimulation of sensory branches of the trigeminal nerve activates a pathway originating at the trigeminal ganglion that leads to antidromic release of neurotransmitters, vasodilation, and increases in CBF. (2) The trigeminal parasympathetic pathway: the sensory afferent from the trigeminal nerve results in parasympathetic vasodilation of the cerebral vasculature via interactions with the facial nerve and the SPG. (3) The central pathway: activation of the rostral ventral lateral medulla (RVLM) induces cerebral vasodilation and increase in mean arterial pressure (MAP), leading to an increased CBF (White et al., 2021).

As for mechanisms by which stimulation of the parasympathetic fibers of the facial nerve increases CBF, the most important is the anatomical connection about the greater superficial petrosal branch of the facial nerve to the terminal SPG, which then projects to the cerebral arteries. Next, connections for the parasympathetic facial nerve to the cerebral arteries other than the SPG likely exist, because the proximal stimulation of the facial nerve trunk produces larger CBF responses than does distal stimulation of the SPG (Borsody and Sacristan, 2016). In addition, stimulation of the facial nerve can also affect the blood supply to the ICA system through the connection with the trigeminal nerve, or affect the blood supply of the vertebral artery system through the connection with the branches of the cervical plexus such as the great auricular, greater, and lesser occipital, and transverse cervical nerves (Diamond et al., 2011). However, its effect is most significant through its own parasympathetic fibers via the SPG.

How does cervical nerve stimulation affect CBF, especially in the posterior circulation system? Although there is evidence indicating parasympathetic innervation of the vertebrobasilar arteries, their origin is still unclear (Roloff et al., 2016). There are hypotheses that innervation of the posterior circulation could originate from the CG (Hardebo et al., 1991; Suzuki and Hardebo, 1991), or the OG. Here the authors postulate the following two potential pathways:

The first is through the connection between the cervical nerve and the trigeminal nerve or facial nerve. There is plenty of evidence to date that shows the convergence of the trigeminal and occipital nerves. For example, the trigeminal cervical complex is such a convergence location (Bartsch and Goadsby, 2003). The overlap between the trigeminal nerve and cervical nerve is known as a convergence mechanism (Piovesan et al., 2003). Because the connection between the V1 of the trigeminal nerve and the cervical nerve through this complex is bidirectional, it is highly likely that stimulation of the scalp in the occipital nerve innervated area may affect CBF through the role of the V1. The connections between the cervical and facial nerves include the connections between the facial nerve and the GON or the LON from C2, and the communication between the facial nerve and the transverse cervical nerve from C2 to C3 (Diamond et al., 2011).

Another possibility is through the connection between the superior cervical ganglion (SCG) and the CmG of the ICA. Stimulation of the GON, LON or the TON from C2 to C3 can communicate with the SCG through the gray ramus communicans. The CmG is a small gangliform swelling, located on the under surface of the ICA, some fibers of which communicate with the trigeminal ganglion, the abducens nerve, and the SPG are distributed to the wall of the ICA (Gray and Lewis, 1918). The SCG is part of the sympathetic nervous system, a division of the autonomic nervous system most commonly associated with the “fight or flight” response. Sympathetic cerebral vasodilation has also been reported to be a compensatory response to vasoconstriction by adrenergic neurons originating in the SCG (Suzuki et al., 2020). Due to the cross talk of parasympathetic and sympathetic fibers on the aforementioned cerebral vascular walls, when stimulating the occipital nerve, the relevant cerebral blood vessels dilate rather than constrict.

Understanding the above mechanisms by which stimulation of different scalp nerves improves CBF not only opens the door to unveil the mechanisms of SA but also helps to elucidate why the effects of stimulating SA points or areas are relatively specific.

## The relative specificity of scalp acupuncture in regulating cerebral blood flow

As of now, many clinical and experimental studies have clearly observed the relative specificity of SA in regulating CBF, which is generally manifested in the following aspects:

(1) The stimulation of the scalp area (SA) has shown significant improvement in CBF compared to stimulation of other parts of the body (body acupuncture) (Hyun et al., 2014; Ratmansky et al., 2016). A study compared the CBF impact of various GV acupoints, and found that effects of Fengfu (GV16), Baihui (GV20), and Dazhui (GV14) on the head and the neck had relatively better effects than other GV points on the trunk. In a similar study, results showed the largest impact or the most significant changes occurred when Fengfu (GV16) was needled (Kim et al., 2018).

(2) Stimulation at any parts of the scalp may improve CBF, but there must exist regional specificity. This is mainly because of the different innervation to the anterior and posterior halves of the scalp. It has been reported that SA with Baihui (GV20) can more significantly affect the ACA and the MCA, while needling Fengfu (GV16) can more significantly affect the PCA (Kim et al., 2018). In other studies, needling Fengchi (GB20) has been shown to improve CBF in posterior (vertebral and basilar) arteries but not the MCAs (Yuan et al., 1998; Im et al., 2014). whereas 20-min of needling at Baihui (GV20) increased CBF and CO2 reactivity in both MCA and ACA (basilar not measured; Byeon et al., 2011). In addition, stimulation of the V1, V2, or V3 of the trigeminal nerve also had different effects on CBF (White et al., 2021). Because the majority of the intracranial blood vessels and dura are innervated by the V1 of the trigeminal nerve, stimulation of SA areas or acupoints innervated by the V1 may have a more broader effect on rCBF. For the characteristics that acupuncture can improve CBF and stimulate different acupoints in different parts of the body to induce different cerebrovascular reactions, some researchers call it “acupoint-cerebrovascular specificity” (Sun et al., 2019).

On the other hand, due to the overlap or convergence of different innervation in many parts of the scalp, stimulation at these overlapping areas may also induce a broader or more significant improvement in CBF compared to stimulation of a single innervated area. This may be the neural characteristic of SA core points or areas frequently used for brain disease. However, it may also be a reason resulting in weakening the regional specificity of SA on CBF. For example, due to the functional connectivity or convergence between the trigeminal and occipital nerves innervating the anterior and the posterior parts of the scalp, respectively (Busch et al., 2006), the effect of stimulating the scalp areas innervated by the trigeminal nerve or GON, ION on CBF may extend to the cerebral arteries innervated by other connecting nerves. It has been reported that needling Baihui (GV20) affected the CBF of the posterior circulation system (Dong et al., 2020), and that needling Fengchi (GB20) and Dazhui (GV14) could improve the CBF of the ICA and MCA in PD patients (Yang et al., 2021).

(3) Stimulating only on a single side of the scalp nerve has an instant effect on CBF on both sides. In a study, Chen and Wu (2011) applied SA in the anterior oblique parietotemporal line on either the affected or the unaffected side for 30 stroke patients, there was an increased CBF bilaterally, but later it was found there was no statistically difference in CBF between SA on the affected and the unaffected side.

(4) The degree of improvement in CBF is related to stimulation parameters (intensity, frequency, duration, etc.) of SA. Although it has been reported that needling the scalp could improve CBF even without repeatedly manipulation of the needles, for example, when the needles were inserted at Fengchi (GB20) and Dazhui (GV14) clockwise to a depth of 15–20 mm, twirled at a angle, and retained for 30 min, an improvement in CBF in ICA and MCA could be observed (Yang et al., 2021), traditionally, SA usually emphasizes the need for repeated rapid rotation or lifting-thrusting of needles during treatment (Wang, 2019). It was reported that when SA was used to treat patients with PCIV, a longer treatment course (sessions) and stronger stimulation (intensity) are more effective in improving vertebrobasilar blood flow velocity and clinical efficacy (Li et al., 2022).

It was observed that SEA of the V1 of the trigeminal nerve to a level of 100 Hz, using 0.2-mA, stimulation enhances CBF of the prefrontal cortex more strongly than 0.1 mA. The intensity of the SEA was set so that no pain was felt but the stimulus was perceptible (Suzuki et al., 2020). In the electromagnetic stimulation experiment of the facial nerve, it was observed that the frequency of 10 Hz could most effectively improve the CBF (Borsody and Sacristan, 2016).

In summary, the effect of SA therapy on improving CBF has both specificity and relativity, the extent of which is determined by the innervated nerves and their density or overlap at the stimulated site, as well as the parameters of the stimulation. The relative specificity of SA points or areas affecting the cerebral vascular system or improving CBF may explain why there is the similar effectiveness of multiple SA schools using different models of SA areas in treating brain diseases. Of course, the presence of some frequently applied core SA points or areas, such as those primary lines described in the international standard of SA are still rational. However, selecting optimal stimulation locations of SA should be based on the relative specificity in future clinical trials.

## Clinical significance of elucidating mechanisms of scalp acupuncture

In clinical practice, elucidating the mechanisms of SA improving CBF would be significant, at least in terms of the following aspects: First, indications of SA therapy shall be clarified. All brain disease patients with reduced CBF would be candidates of SA therapy, no matter if they are acute or chronic, ischemic or hemorrhagic. Second, selection and determination of core SA points or areas for various brain diseases shall be based on a solid scientific foundation. The optimal SA points or areas for brain disease shall be on the locations innervated by overlapping nerves, which would improve rCBF. Third, changes to CBF, especially rCBF can be a quick objective marker used to assess instant or long-term therapeutic effects, or to determine the proper stimulation parameters of SA.

In short, such clinical strategies based on the effect of improving CBF will further enhance the efficacy of SA for brain diseases, as well as to avoid SA research pitfalls based on the misconceptions of SA points or areas corresponding to the cortical functional zones.

## Conclusion

It has been known that reduced CBF is often associated with the occurrence of many brain diseases, not only acute ischemic strokes, but also serious neurodegenerative diseases including PD, AD, and other forms of cognitive decline. Thus, improving or restoring CBF can prevent and treat these types of diseases theoretically. In fact, presently, there is an adequate amount of evidence-based clinical research (RCTs) that shows SA does have significant therapeutic effects on these brain diseases, especially ischemic strokes, though certain research limitations remained in such RCTs.

Furthermore, stimulating the SA points, areas or nerves innervating the scalp, especially the trigeminal nerve, can significantly improve CBF, which parallels the instant or long-term improvement of clinical symptoms of brain diseases. Neural pathways to improve CBF by stimulation of the trigeminal, facial, or cervical nerves, have also been gradually revealed. The presence of the core SA points or areas frequently used can also be rationally explained from the characteristics of nerve distribution, including nerve overlap or convergence in different parts of the scalp. However, these characteristics that would determine the role of these SA points or areas are relatively specific, and not due to a direct correspondence between the current hypothesized SA points, areas and the functional zones of the cerebral cortex.

Based on the above evidence chain, the efficacy of SA in treating brain diseases, especially ischemic, is mostly achieved by stimulating the nerves innervating the scalp, specifically the trigeminal nerve, to improve CBF. However, the therapeutic role of improving CBF is not always easily comprehended for brain diseases caused by other etiologies. It has been known that mechanisms of SA in treating different brain diseases might also be multifaceted, such as anti-inflammatory, reducing central sensitization, promoting angiogenesis and tissue repair, etc. Among these mechanisms, the anti-inflammatory response by SA may be secondary to the improvement of CBF in the treatment of brain diseases.

There have been a number of studies in which SA has an impact on the local or systemic immune function and inflammatory response (Liu et al., 2012; Wang J. H. et al., 2022). The cholinergic anti-inflammatory mechanism might be activated by trigeminal parasympathetic pathways (such as trigeminal-vagal reflex) during SA. It is entirely possible that SA, while improving CBF, could also arouse the anti-inflammatory reflex, which is critical to those brain diseases with significant inflammation due to brain tissue injury (hemorrhagic stroke, traumatic brain injury, etc.). However, since the anti-inflammatory effect induced by acupuncture occurs slower than the improvement in CBF, the latter seems more suitable to explain the instant effect of SA in the treatment of brain diseases. Moreover, the improvement of rCBF in brain injury also facilitates the recruitment of leukocytes and pro-inflammatory cytokines in the early stages of injury while increasing the concentration of anti-inflammatory cytokines in later stages. Thus, there is an obvious need to further investigate which of these mechanisms are independent of improving CBF or whether they act synergistically, or have different levels of significance in the treatment of various types of brain diseases with SA.

The authors believe that understanding the neural control of SA on CBF is not only key in revealing the underlying mechanism in treating brain diseases, but also helps clarify above-mentioned roles evoked by SA. Moreover, the improvement of CBF by SA can be used as an objective indicator for effective SA stimulation in future studies.

The effort of elucidating this scientific foundation of SA therapy will undoubtedly further advance the clinical applications of SA in treating brain diseases, such as broadening the scope of indications based on its effect on improving CBF; clarifying or expanding the applications of SA; selecting the optimal stimulation points or areas of SA and stimulation parameters based on different conditions and scalp innervation characteristics and establishing a rapid objective indicator based on changes in rCBF to evaluate instant or long-term therapeutic effects. Such clinical strategies will all further improve the efficacy of SA in treating brain diseases and increase its repeatability, as well as avoid the pitfalls of SA research or practice due to misconceptions of SA points or areas corresponding to the cortical functional zones.

## Author contributions

GJ conceptualized the theme of the review and drafted the manuscript. LJ critically reviewed and revised the manuscript. BJ performed the literature search and drew the figure. JZ and BH reviewed and edited the manuscript. SL helped with the final revision of the manuscript. All authors approved the final version of the manuscript.
